# “I can’t tell whether it’s my hand”: a pilot study of the neurophenomenology of body representation during the rubber hand illusion in trauma-related disorders

**DOI:** 10.3402/ejpt.v7.32918

**Published:** 2016-11-21

**Authors:** Daniela Rabellino, Sherain Harricharan, Paul A. Frewen, Dalila Burin, Margaret C. McKinnon, Ruth A. Lanius

**Affiliations:** 1 Department of Psychiatry, University of Western Ontario, London, ON, Canada; 2 Department of Neuroscience, University of Western Ontario, London, ON, Canada; 3 Department of Psychology, University of Western Ontario, London, ON, Canada; 4 Imaging Division, Lawson Health Research Institute, London, ON, Canada; 5 SAMBA (SpAtial, Motor & Bodily Awareness) Research Group, Psychology Department, University of Turin, Turin, Italy; 6 Mood Disorders Program, St. Joseph’s Healthcare, Hamilton, ON, Canada; 7 Department of Psychiatry and Behavioural Neurosciences, McMaster University, Hamilton, ON, Canada; 8 Homewood Research Institute, Guelph, ON, Canada

**Keywords:** Body ownership, dis-embodiment, multi-sensorial integration, autonomic arousal, depersonalization, derealization, consciousness

## Abstract

**Background:**

Early traumatic experiences are thought to be causal factors in the development of trauma-related dissociative experiences, including depersonalization and derealization. The rubber hand illusion (RHI), a well-known paradigm that measures multi-sensorial integration of a rubber hand into one’s own body representation, has been used to investigate alterations in the experience of body ownership and of body representation. Critically, however, it has never been studied in individuals with trauma-related disorders.

**Objective:**

To investigate body representation distortions occurring in trauma-related disorders in response to the RHI.

**Method:**

The RHI was administered to three individuals with the dissociative subtype of posttraumatic stress disorder (PTSD), and subjective, behavioral, cardiovascular and skin conductance responses were recorded.

**Results:**

Participants’ subjective experiences of the RHI were differentiated and complex. The illusion was induced following both synchronous and asynchronous brushing and variably evoked subjective distress, depersonalization and derealization experiences, tonic immobility, increased physiological arousal and flashbacks.

**Conclusions:**

The present findings point towards the RHI as a strong provocation stimulus that elicits individual patterns of symptom presentation, including experiences of distress and dissociation, in individuals with trauma-related disorders, including the dissociative subtype of PTSD.

**Highlights of the article:**

- The rubber hand illusion (RHI) elicits distress, tonic immobility, depersonalization and derealization, and autonomic responses in individuals with trauma-related disorders, including the dissociative subtype of posttraumatic stress disorder (PTSD).
- RHI effects related to body misrepresentation may trigger altered experiences related to body ownership.
- The RHI represents a promising paradigm for studying the neurophenomenology of body distortion in individuals experiencing trauma-related altered states of consciousness (TRASC).

Dissociation, frequently associated with psychological trauma (Brand, Loewenstein, & Spiegel, 2013; Dalenberg et al., 2012), describes the experience of psychological detachment from normal perceptions of one’s self or surroundings resulting in an altered state of consciousness (Diagnostic and Statistical Manual of Mental Disorders-5 [DSM-5], American Psychiatric Association [APA], 2013). In recognition of this strong association between dissociation and trauma, a dissociative subtype of posttraumatic stress disorder (PTSD), focusing on the presence of symptoms of depersonalization and derealization was recently added to the DSM-5 (Lanius, Bethany, Vermetten, Frewen, & Spiegel, 2012). Frewen and Lanius (2015) described trauma-related dissociation and altered states of consciousness in the context of a 4-dimensional model (“4-D model”) that classifies symptoms of trauma-related psychopathology into those that occur within normal waking consciousness versus those that are intrinsically dissociative and associated with trauma-related altered states of consciousness (TRASC) across four dimensions: one’s experience of (1) time, (2) thought, (3) body and (4) emotion (also see Lanius, 2015).

Traumatized individuals that experience TRASC related to the experience of their body are described by the 4-D model as experiencing depersonalization symptoms, specifically, those involving partial or full disembodiment (Frewen & Lanius, 2015; Sierra & Berrios, 1998). Partial disembodiment occurs when one experiences a disconnection between particular parts of one’s body and the greater gestalt of one’s embodied sense of self, such as in limb disownership and paralysis. Such disowned body parts are thus experienced as “non-self,” and are often described as feeling “strange” or “different from expected” (Frewen & Lanius, 2015). By contrast, in cases of full disembodiment, one feels disconnected from the whole of one’s body, as in an out-of-body experience (Blanke & Metzinger 2009; Frewen & Lanius, 2015). Although the 4-D model focuses on TRASC, depersonalization symptoms can also be observed in psychiatric and neurologic disorders outside of a trauma context. For example, depersonalization/derealization disorder, migraines and seizures are frequently associated with altered bodily representation (Alper et al., 1997; APA, 2013; Baker et al., 2003; Blau, 1992; Kenna & Sedman, 1965).

The rubber hand illusion (RHI; Botvinick & Cohen, 1998) is a well-described experimental paradigm that can be used to invoke alterations in the experience of the body schema through simultaneous visual and proprioceptive feedback (Ehrsson, Holmes, & Passingham, 2005; Longo, Schüür, Kammers, Tsakiris, & Haggard, 2008). Here, the participant is instructed to stare at a rubber hand while his/her actual hand is hidden from view during simultaneous brushing of both the rubber and actual hand. This manipulation can create a temporary distortion in the physical schema of the body, with a subjective feeling of ownership of the rubber hand in approximately 70–80% of tested participants, where participants perceive the rubber hand as their own (Botvinick & Cohen, 1998; Ehrsson, Spence, & Passingham, 2004). Accordingly, the RHI serves as an important experimental paradigm to study the neurophenomenological bases of the experience of conscious embodiment, allowing for investigation of the correlation between psychological identity and one’s sense of body ownership (Longo & Haggard, 2012).

To date, no studies have examined response to the RHI among individuals with trauma-related disorders and dissociative symptoms. Here, we provide RHI case reports of three traumatized patients with a diagnosis of the dissociative subtype of PTSD with comorbid dissociative disorder not otherwise specified (DDNOS). We hypothesized that, given their chronic tendency toward experiences of TRASC of their body and the high likelihood of the RHI inducing changes in body representation, these participants might readily experience a pronounced RHI. We also examined whether experiences of the RHI might lead to other forms of dissociation and TRASC (e.g., derealization symptoms and TRASC of time, thought and emotion) and measured arousal and perceived distress through self-reports and physiological recordings (skin conductance response, heart rate and heart-rate variability).

## Ethics

This study was approved by the local research ethics board. All names have been changed to protect patient privacy. All patients consented to their inclusion in the report and had the opportunity to review the manuscript prior to its submission.

## Methods

### Psychological assessment

All patients were diagnosed with PTSD based on the *Clinician-Administered PTSD Scale* (CAPS) (cut-off for PTSD ≥50; Blake et al., 1995). The *Structured Clinical Interview for Dissociative Disorders* (SCID-D; Steinberg, Cicchetti, Buchanan, & Hall 1993) was conducted to assess dissociative disorders, and the *Structured Clinical Interview for DSM-IV* (SCID-I; First, Spitzer, Gibbon, & Williams, 2002) was administered in order to document any other current Axis I psychiatric disorders. Prior to the experiment, the patients were also administered a battery of questionnaires:

- The Scale of Bodily Connection (SBC) (Price & Thompson, 2007) was used to assess bodily awareness on a Likert scale from 0 (not at all) to 4 (all of the time).
- The Dissociation Tension Scale (DSS-4) (Stiglmayr, Schmahl, Bremner, Bohus, & Ebner-Priemer, 2009) was used to compare the patient’s perception of dissociative states associated with bodily consciousness and psychological identity, based on a Likert scale of 0 (none) to 9 (very strong).
- The PTSD Checklist for DSM-5 (PCL-5) (Weathers et al., 2013) and appended items assessing TRASC (Frewen, Brown, Steuwe, & Lanius, 2015) were administered to assess state PTSD and dissociative symptoms on a Likert scale of 0 (not at all) to 4 (extremely).

See Tables 1–3 for a summary of obtained scores on these measures.

**Table 1:** Psychometric assessments

Psychometric assessments	Stephanie		Dawn		Michelle	
CAPS total score	61		99		68	
Dissociative subtype^a^	Yes		Yes		Yes	
SCID/D	DDNOS – in partial remission		DDNOS		DDNOS – in partial remission	
SBC^b^	2.86		1.83		2.9	
Tension rating^c^	Pre=7	Post=N/A^d^	Pre=9	Post=9	Pre=8	Post=3
DSS-4^e^ (mean)	Pre=4.25	Post=N/A^d^	Pre=5	Post=6.75	Pre=0	Post=0
State PCL-5 TRASC (sum scores)	N/A	N/A	Pre=22	Post=23	Pre=4	Post=3
SCID-I	– past major depressive disorder		– recurrent major depressive disorder – trauma-related eating disorder		– undifferentiated somatoform disorders – current agoraphobia without panic – past recurrent major depressive disorder – past alcohol and substance dependence – past panic disorder with agoraphobia	

a Assessed through CAPS, depersonalization and derealization items (score≥4, frequency+intensity)

b general population average score=3.645±0.645 (Price & Thompson, 2007)

c measured by the DSS-4, item on state tension

d the participant was not able to fill the DSS-4 after the task

e healthy population range=0<DSS-4<1 (Barnow et al., 2012). CAPS: Clinician Administered PTSD Scale; DDNOS: dissociative disorder not otherwise specified; DSS-4: Dissociation Tension Scale–short version; PCL-5: PTSD Checklist for DSM-5; pre: pre-task; post: post-task; SBC: Scale of Bodily Connection; SCID-D: Structured Clinical Interview for Dissociative Disorders; SCID-I: Structured Clinical Interview for DSM-IV.

**Table 2:** Pre- and post-task scores of trauma-related altered states of consciousness for Dawn and Michelle

	Dawn		Michelle	
		
State PCL-5 TRASC*	Pre	Post	Pre	Post
#1: Flashbacks of traumatic events	3	3	0	2
#2: Altered sense of time	3	3	0	0
#3: Marked loss of emotional feeling	2	4	0	0
#4: Feeling what you are experiencing is not real	3	3	0	0
#5: Out of body experience	2	2	0	0
#6: Feeling like a part of your body is not your own	2	2	0	0
#7: Identity confusion	1	2	1	0
#8: Divided or multiple senses of self	3	2	3	1
#9: Loss of time	3	2	0	0
#10: Hearing voices	0	0	0	0

* Examples of questions:

#1: “Do you feel as if a traumatic event from the past is happening in the present? If so, do you feel like you are reliving the event rather than only remembering it?”

#2: “Do you feel as if you have little sense of the passage of time? Or do you feel as if time has slowed down, speeded up, or seems like it is stopped or standing still?”

**Table 3:** Pre- and post-task scores of the DSS-4

	Stephanie		Dawn		Michelle	
			
DSS-4	Pre	Post	Pre	Post	Pre	Post
#1: Current inner tension.	7	N/A	9	9	8	3
#2: My body or parts of my body do not belong to me.	7	N/A	2	7	0	0
#3: I have problems hearing, for example, I hear sounds that are close to me as if they are coming from far away.	3	N/A	6	7	0	0
#4: I have the impression that other people, objects, or the world around me are not real.	7	N/A	6	7	0	0
#5: I have the impression that my body or a part of it is insensitive to pain.	0	N/A	6	6	0	0

### Physiological measurements

The Nexus-10 & Bio Trace+ physiological sensors technology (MindMedia b.v., The Netherlands; Kurumbanshi, Kapur, & Bajaj, 2014) was utilized to obtain heart rate variability (HRV) from a finger blood-volume pulse sensor, and finger galvanic skin conductance (GSC) was also acquired using the same system. Two measures of HRV were calculated using BioTrace software: the standard deviation of interbeat intervals (SDNN), which has shown to be an indicator of overall HRV (Guédon-Moreau et al., 2012; Saul, Albrecht, Berger, & Cohen 1987; Tan, Wang, & Ginsberg, 2013), and the root mean square differences in interbeat intervals (RMSDD), which is thought to be an indicator of vagal outflow and thus reflects parasympathetic nervous system activation (PNS; Bigger et al., 1989; Kleiger, Stein, & Bigger, 2005). By contrast, GSC is a measure of sweat gland activity and is thought to be indicative of sympathetic nervous system (SNS) arousal (Darrow, 1936; Montagu & Coles, 1966). In order to observe baseline physiological measures, a 2-min resting state assay was performed prior to the experiment. When analyzing HRV data, artifacts were both automatically (using a ±20% difference based on the previous interbeat interval) and manually (via visual inspection) removed to account for environmental and biological noise (Xu & Schuckers, 2001).

Please see Fig. 3 for a summary of physiological measures obtained.

### RHI procedure

Following standard methods (Burin et al., 2015), the RHI paradigm consisted of a black box (60 cm×40 cm×20 cm) with a perpendicular panel dividing it in half (30 cm×40 cm×20 cm), arranged such that the patient’s real hand would be hidden and only the life-like rubber hand would be within the patient’s view. The patient wore a white cape on her body to ensure that only the rubber hand was in view and was oriented such that the patient’s shoulder was in line with the rubber hand. Both the real and rubber hand were positioned with fingers pointing forward and palms facing down, with a distance between the real and the rubber hand of approximately 15 cm.

For the illusion experiment, the right hand (dominant hand for all three subjects) was brushed with a paintbrush for two trials, each lasting 2 min, as described by Costantini and Haggard (2007). Only one hand was tested as this procedure induced depersonalization and derealization symptoms in some participants during pilot testing of the current study. During the first trial, the administrator initiated asynchronous brushing, alternately brushing the index fingers of the rubber hand and the actual hand. The second trial consisted of synchronous brushing where the administrator simultaneously brushed the index fingers of both the rubber hand and the actual hand. The trial order was alternated between participants.

**Fig. 1:**
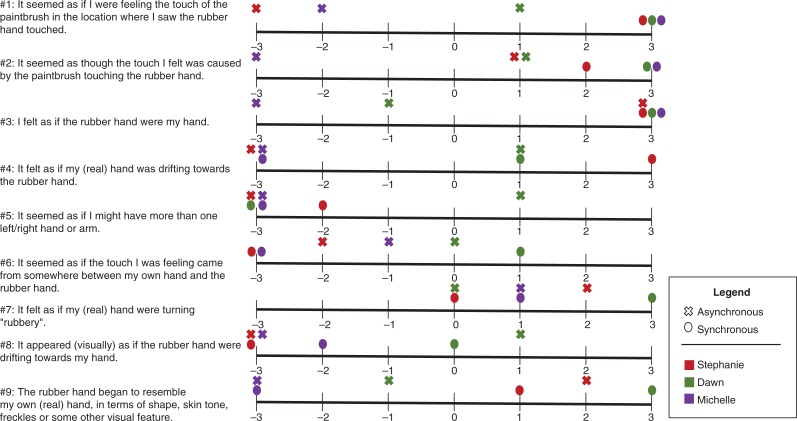
Answers from all three case report subjects to the nine-question post-trial questionnaire administered following each asynchronous and synchronous trial. Each question is evaluated on a Likert scale spanning from – 3 (complete disagreement) and+3 (complete agreement). The first three questions assess the illusion effect, whereas the last six serve as control questions. This plot reveals that all subjects endorsed the subjective perception of the illusion following the synchronous trial, whereas only Stephanie endorsed the perception of the illusion following the asynchronous trial. It is important to note that for both Stephanie and Dawn, the asynchronous trial was performed first and the synchronous trial second, whereas for Michelle, the order was reversed.

**Fig. 2:**
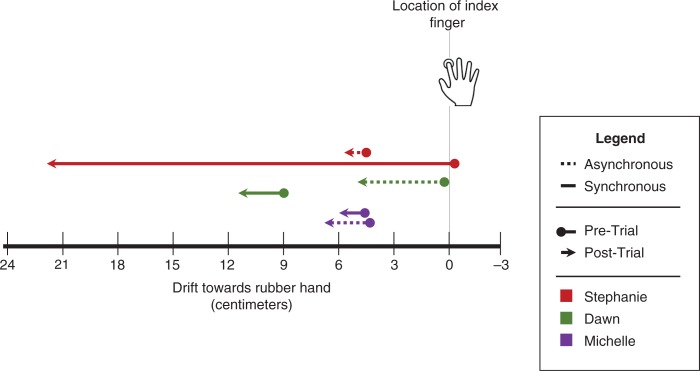
The diagram depicts the participant’s objective perception of the rubber hand illusion, as it shows the participant’s perception of her real index finger location relative to the location of the rubber hand both before and after the asynchronous and synchronous brushing trials. The lines for each participant are presented in chronological order as both Stephanie and Dawn received the asynchronous trial first, whereas Michelle received the asynchronous trial second. All participants perceived a drift towards the rubber hand after both asynchronous and synchronous brushing trials.

Prior to each trial, the box was covered with a flat lid, and a ruler (in centimeters) was placed on the lid. The participant was then asked to report the number on the ruler that corresponded to her perceived index finger (Burin et al., 2015). This procedure was repeated six times, changing randomly the position of the ruler each time. The same exercise was performed after the trial to determine proprioceptive drift (see Fig. 2 for pre- and post-trial individual proprioceptive drift). The absolute drift was obtained by subtracting the average post-trial estimations from the average pre-trial estimations for each subject (Botvinick & Cohen, 1998). A nine-item questionnaire created from Botvinick and Cohen’s (1998) original RHI study was administered verbally to the patient after each trial to identify the subjective perception of the illusion (see individual scores in Fig. 1). The DSS and the PCL-5 were administered before and after each trial to assess state dissociative symptoms.

### Case report A: Stephanie

#### History and diagnosis

Stephanie is a 50-year-old woman who experienced emotional abuse from a young age. Stephanie’s father was largely absent due to alcoholism while her mother was emotionally neglectful. She was sexually abused by her brother and was repeatedly gang-raped as an adolescent. As an adult, Stephanie spent nearly 8 years as a psychiatric inpatient setting before she was referred to a trauma specialty service with a diagnosis of PTSD (dissociative subtype), a DDNOS and major depressive disorder. During previous treatment, Stephanie described depersonalization experiences involving a lack of body ownership of her hands, for example, during mindful body-scan exercises. She also described out-of-body experiences that occurred during recall of traumatic memories. After undergoing trauma therapy for several years, however, Stephanie no longer met criteria for PTSD, DDNOS and depression, and her overall level of functioning improved significantly. Indeed, over the past 4 years, Stephanie had worked productively as a teacher in Canada and more recently abroad. Unfortunately, however, she experienced a relapse of PTSD and depersonalization and derealization symptoms several months prior to the present RHI assessment upon witnessing a grandfather physically abusing his grandson and sought further short-term treatment.

#### Observations during RHI

Prior to the experiment, Stephanie reported an experience of high inner tension and dissociative symptoms related to body disownership and derealization on the DSS-4 (see Table 3). The intensity of these experiences increased, however, during administration of the RHI. Indeed, simply placing the rubber hand within Stephanie’s view caused immediate anxiety, where she commented “It looks real” and pointed out its resemblance to her own hands during experiences of partial disembodiment: “[It] looks like when my hands are detached … it looks like it’s detached from my body.” Following the first experimental trial with asynchronous brushing, the RHI was induced, where Stephanie described “This is kind of bizarre” and that, when her actual hand was stroked, it felt as if the experimenter “was stroking this one [the rubber hand] … and the thing moves [the rubber hand].” She noted further that “I don’t like that it moves. It’s … It’s like it’s attached itself to me.” Stephanie related that “I can’t tell whether it’s my hand or somebody else’s hand, or who it belongs to.” Interestingly Stephanie further described the experience as “like being dissociated” and that she experienced an associated feeling of distress accompanied by upset stomach. She also indicated that she was “losing focus” and noted some derealization symptoms: “I’m having trouble figuring out what’s real and what’s not.” She indicated further that her real hand felt like it was turning rubbery and that the rubber hand began to resemble her own hand. She did not, however, report feeling like she had more than one right hand. The results from the measurements indicated that she experienced a slight proprioceptive drift of her real hand toward the rubber hand (post-trial>pre-trial, *M*=1.1±0.68 cm; see Fig. 2).

**Fig. 3:**
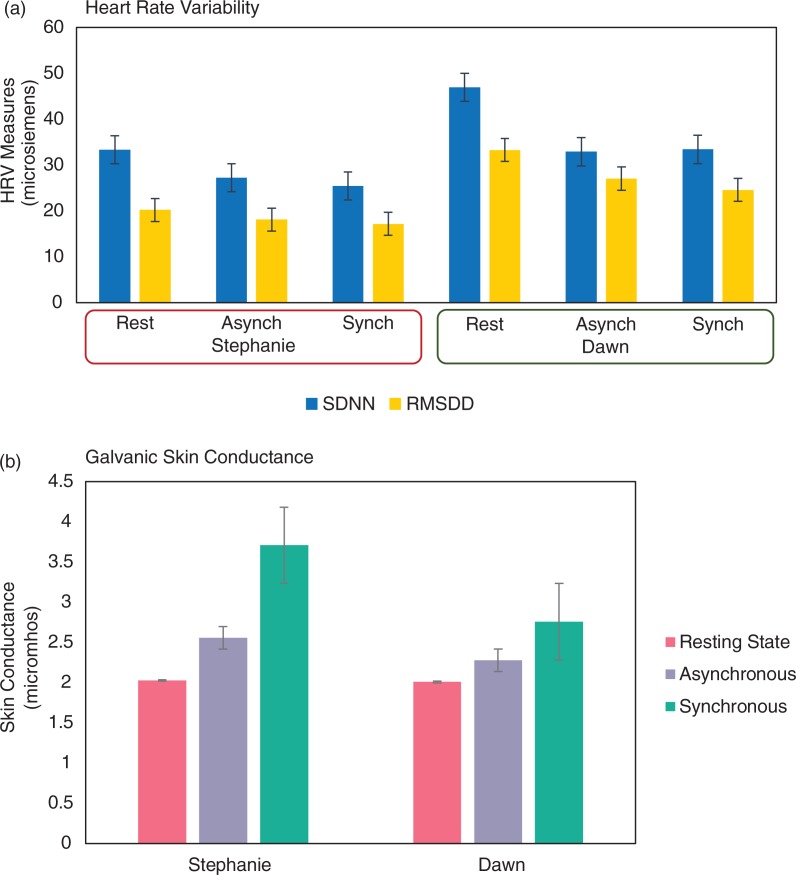
Physiological data. The graph depicts the heart rate variability (left *y*-axis) and galvanic skin conductance (GSC) obtained for both Stephanie and Dawn during each stage of the RHI experiment. (a) Standard deviation of interbeat intervals (SDNN) is an overall indicator of total HRV, whereas root mean square of interval differences (RMSDD) is an indicator of vagal outflow, a proxy for parasympathetic activity. Both Stephanie and Dawn experienced overall decreases in SDNN and RMSDD during the RHI experiment. Stephanie experienced gradual declines in both SDNN and RMSDD. Dawn showed higher SDNN and RMSDD values than Stephanie but still showed a gradual decline in RMSDD. Dawn experienced a sharp decrease from rest in SDNN after the asynchronous (asynch) trial, but instead showed a slight increase after the synchronous (synch) brushing. (b) GSC is an indicator of sympathetic arousal, as it measures sweat gland activity. In both Stephanie and Dawn, there was a progressive increase in skin conductance throughout the experiment. In Stephanie, there was a significantly greater increase following the synchronous trial when compared to the increase observed after the asynchronous trial.

After the second trial with synchronous brushing, Stephanie reported a further increase in severity of depersonalization/derealization symptoms. She indicated “I don’t know where I am” and showed explicit cognitive slowing (long pauses, difficulty in understanding questions). At this time, Stephanie experienced a flashback of a traumatic rape dating back to 1978 when she was an adolescent. She indicated further that during the RHI “her real hand jumped through the box and became the rubber hand … it did not drift, it flew […] just all of a sudden it was like the two came together and that was it.” This experience of partial body depersonalization was again associated with anxiety: “anxiety … couldn’t figure out what was going on … knowing that my hand should be there [under the box] but it’s here [where the rubber hand is] and how could they both be there?” This observation was consistent with the measures of the perceived location of her real hand’s index finger following the trial, indicating that she experienced the RHI rather strongly (post-trial>pre-trial, *M*=22.83±4.21 cm; see Fig. 2). Consistent with this, during the post-synchronous trial estimation, when asked to estimate where her actual index finger was located, for the first three estimations, her eye-gaze directed towards the rubber hand, while for the last three estimations she initially directed her eye-gaze alternatively to her own hand and the rubber hand, and finally provided the estimations while looking towards the rubber hand’s location. Interestingly, Stephanie also indicated that over the period of anxiety provoked by the RHI, she experienced tonic immobility: “You can’t move, there’s nothing you can do to make it stop … I think there is also the fear that if you try, something will happen, so you’ve just got to let it play out.” Finally, Stephanie showed a progressive decrease in HRV (both for the SDNN and RMSDD) and a progressive increase in GSC, suggestive of decreases in PNS activity and increases in SNS activity (see Fig. 3).

### Case report B: Dawn

#### Patient history and diagnosis

Dawn is a 64-year-old woman who was adopted at age three into a household with an absent father and an emotionally abusive mother that neglected her and made her feel inferior to her siblings. Her adoptive mother repeatedly told Dawn that she wished Dawn had never been born and never existed. Eventually, Dawn was referred to a trauma specialty clinic after being diagnosed with what were considered intractable symptoms of PTSD (dissociative subtype) and depression related to her childhood abuse. She presently experiences a reduction in her symptoms of PTSD, depersonalization, derealization, and depression and works in the mental health field.

#### Observations during RHI

Prior to the RHI, Dawn reported a high inner tension but comparably few disembodiment symptoms on the DSS-4 (Table 3). She did, however, report difficulty identifying and expressing emotions as well as perceiving bodily sensations related to her emotional state on the SBC (Table 1). On the SBC questionnaire administered prior to the experiment, Dawn indicated that she often feels frozen or numb during uncomfortable situations, and that it was difficult to listen to information from her body related to her emotional state. Her overall poor body awareness was therefore considered a potential susceptibility factor for the RHI (Tsakiris, Tajadura-Jiménez, & Costantini, 2011).

As was the case with Stephanie, Dawn experienced distress and dissociation immediately upon seeing the rubber hand, reporting that she was having “difficulty staying present.” This increased during the asynchronous trial, which Dawn described as “kind of disconnecting … Part way through it was harder to figure out which [hand] was which.” Interestingly, Dawn was able to attribute her experience of “disconnection” to a dissociation between visual and tactile perception: “it was more difficult to actually feel it than it was to see it. Um, there was a disconnect there.” Behavioral testing confirmed Dawn’s experience of a proprioceptive drift towards the rubber hand after the asynchronous trial (post-trial>pre-trial, *M*=5.33±2.42 cm; see Fig. 2). However, during administration of the post-trial questionnaire, the subjective strength of the illusion was closer to neutral scores, all within the range −1 to +1 on the Likert scale (Fig. 2). Sensing her discomfort, the interviewer asked how she was feeling and, like Stephanie, Dawn described an experience of tonic immobility, specifically stating that her body felt frozen and unable to move. She also experienced a difficulty “staying present” that she attributed to a difficulty having to “stay in one position” during the experiment. Asked if she felt she was unable to move she described a sensation: “Kind of from the neck down.” Following administration of the synchronous trial, Dawn experienced an intensified fear response that she attributed to the RHI, specifically, her hand “taking on the form of the rubber hand … I guess that it seems so real.”

Critically, Dawn continued to experience tonic immobility through the duration of the session, to the point of requiring assistance removing the experimental dress (white cape) at the conclusion of the experiment. She reported later that she could only feel her fingertips throughout the experiment, but not her full hands, and she was observed to press down on her hands with her fingertips, an action she reported served to “fight the disconnection and try to stay present.” Interestingly, during debriefing, she also noted that her sense of time and space had become altered during the experiment, which she thought lasted only 10–15 min whereas the session actually required approximately 90 min to complete. She also reported that the partial disembodiment she experienced in her hand prompted a vulnerability to a full (whole body) out-of-body experience: “There were points [in time] I could feel the out-of-body stuff, which I don’t feel very often. I had to fight the urge to ‘leave’ [my body].”

The experimenter administered the post-trial estimations of index finger location, and they demonstrated a proprioceptive drift towards the rubber hand (post-trial>pre-trial, *M*=2.5±0.84 cm; see Fig. 2), although lower than after the asynchronous trial. The pre-measurements were already closer to the rubber hand, carrying over from the asynchronous trial, suggesting that Dawn did not come back from her drift experienced during the asynchronous trial. On the post-trial questionnaire (Fig. 1), however, Dawn this time reported a stronger subjective perception of the RHI. Like Stephanie, Dawn also explicitly reported the feeling of the rubber hand as becoming her own right hand, instead of experiencing having more than one right hand. According to the DSS-4 (Table 3), she continued to maintain a high inner tension but felt more disconnected from her body parts. Relative to her autonomic responses, Dawn experienced an overall decrease in HRV, including for SDNN and RMSDD, in conjunction with a progressive increase in GSC (Fig. 3).

### Case report C: Michelle

#### Patient history and diagnosis

Michelle is a 59-year-old woman who experienced multiple instances of childhood physical and sexual abuse occurring between the ages 4 and 18, and who also grew up with a physically and emotionally abusive and neglectful mother. Michelle was also married to an emotionally, physically and sexually abusive man for over 25 years. Michelle has had over 50 psychiatric admissions, lasting from 3 days up to 6 months. She was diagnosed with PTSD (dissociative subtype), DDNOS (in partial remission), major depressive disorder (recurrent) and dysthymia. Her symptoms have become more stable in recent years, and she has not had any psychiatric admissions for 5 years.

#### Observations during RHI

Michelle reported high inner tension but no disembodiment symptoms on the DSS-4 prior to the experiment (Table 3). On the PCL-5 and TRASC items (Frewen et al., 2015), she indicated feeling “a little bit” of identity confusion (associated with an unstable sense of self) and an experience of multiple, divided senses of self “quite a bit” (see Table 2), findings consistent with her diagnosis of DDNOS.

Due to technical difficulties, physiological data were not collected for this case.

Upon being presented with the rubber hand, Michelle experienced an immediate sense of anxiety, commenting: “It’s a little scary, kind of looks a little too real, but kind of feels like it should be my hand there but it’s not … just makes me feel a little shaky … a bit like a dead person’s hand.”

In contrast to the protocol order performed in Case Report A and B, for this case we decided to administer the synchronous trial first. After the synchronous trial, Michelle described her experience of the RHI as accompanied by distress: “I want to say scary, but that’s not maybe the word … It felt like it was my other hand … it just kind of gave me the creeps.” Her proprioceptive drift (post-trial>pre-trial drift) towards the rubber hand (*M*=1.40±2.51 cm; see Fig. 2) indicated she had, to some degree, experienced the illusion. Consistent with this, she endorsed many accompanying subjective perceptions of the illusion on the post-trial questionnaire (Fig. 1). Interestingly, when she was asked if the rubber hand began to resemble someone else’s hand, she stated that it reminded her of her deceased mother’s hand. Incidentally, she also noted that, unbeknownst to the investigators, the testing day occurred on the 1-year anniversary of her mother’s death.

Following the asynchronous trial, Michelle’s responses to the post-task questionnaire suggest a less pronounced RHI (see Fig. 1). However, she continued to perceive the rubber hand as resembling her mother’s hand. Interestingly, the difference between pre- and post-trial proprioceptive drift indicated a *larger* drift towards the rubber hand in the asynchronous trial, as compared to the synchronous trial (post-trial>pre-trial drift, *M*=2.50±2.40 cm; see Fig. 2).

Michelle also commented generally that the experimental session provoked an increased experience of flashbacks (see Table 2). This is particularly interesting given Michelle’s report that the rubber hand resembled that of her deceased mother, a former perpetrator of her past traumas. As such, the representation of Michelle’s own bodily space, when perturbed by the illusion, may have been experienced as being invaded by her perpetrator. Accordingly, her experience that the hand was her own *and* the perpetrator’s may have triggered the flashbacks and distress she experienced during the experiment. It is noteworthy, however, that Michelle’s reaction to the RHI may be related to the anniversary of her mother’s death. Future studies involving larger samples are therefore warranted.

## Summary and conclusions

Here, we described responses to the presentation of the RHI among three individuals with the dissociative subtype of PTSD (D-PTSD) and comorbid DDNOS. The evidence reviewed here strongly suggests that, as a provocation stimulus, the RHI has the potential to serve as a potent elicitor of distress, depersonalization, derealization, tonic immobility and dissociative flashbacks in persons with D-PTSD. As expected, the RHI is most pronounced during synchronous relative to asynchronous brushing, suggesting its partial mediation through divergent multisensory perception. Nonetheless, asynchronous brushing and indeed mere presentation of the rubber hand was seen to variably evoke subjective distress and depersonalization and derealization experiences across cases, including an explicit case of traumatic reminding in Michelle, suggesting that its mechanism of action may be more multifaceted and complex in traumatized individuals than is observed in healthy participants. Notably, although the asynchronous trial is generally regarded as a control trial in RHI experiments, Longo et al. (2008) suggested that this type of brushing can lead to *deafference*, where conflicting sensory information may lead to restricted transmission of this input into the brain. Given that mind-body connections appear more vulnerable to disruption in traumatized patients with a history of depersonalization symptoms, conflicting sensory information may predispose these patients to experiencing feelings of disembodiment during the RHI paradigm (Frewen & Lanius, 2015).

In this vein, we would like to underline that each patient made it explicitly clear that they perceived the rubber and their actual hands as “coming together.” Here, it is interesting to note that results of previous studies investigating the RHI in healthy participants (Moseley, Gallace, & Spence, 2012) suggested that the rubber hand substitutes the actual hand of the participant in the neural representation of one’s own body schema. Indeed, Moseley et al. (2012) proposed that during the illusion effect, the cortical representation of the space pertaining to one’s own hidden hand becomes occupied by the space containing the rubber hand. As a result, homeostatic control and sensory processing from the previous space decreases, as shown by drops in temperature of the real hidden hand (Moseley et al., 2008). Future studies in PTSD should therefore also explore changes in temperature within the real hand during the RHI.

The phenomenology of the RHI experienced by traumatized individuals appears differentiated and complex. Both Stephanie and Dawn reported significant depersonalization and derealization symptoms that were accompanied by distress and tonic immobility following the RHI, which, in turn, were associated with reduced PNS and increased SNS arousal. We also found that the RHI may serve as a potent elicitor of trauma-related flashbacks-re-experiencing (Stephanie and Michelle). Finally, in PTSD patients with prominent dissociative experiences, we found that the bodily misperception provoked by the RHI can further alter other dimensions of subjective consciousness, including time perception (Stephanie) and identity.

Here, we hypothesize that, among PTSD patients with dissociative symptoms who do not present with prominent identity confusion and fragmentation, the RHI, at most, induces a relatively transient state of partial disembodiment (depersonalization). However, in the presence of significant identity disturbance, the RHI may also occasion significant ego dissolution, prompt traumatic re-experiencing, and lead to more marked distress. Further neurobiological investigation of the RHI in complex dissociative disorder patients is therefore warranted to increase our understanding of the neural mechanisms underlying the conscious embodiment of human self and its vulnerability to alteration through experimental manipulation and exposure to traumatic stress. In addition, future research that includes a comparison group and larger samples is warranted. The cases presented here offer further clinical insight into how consciousness can be compromised when mind and body are disconnected and emphasizes the importance of developing specific psychological treatments for those that experience TRASC.
